# Evaluation of the conserved subunit of the pathogenic *Leptospira* FlaB protein-based immunochromatographic test for the diagnosis of acute leptospirosis

**DOI:** 10.1128/spectrum.00307-25

**Published:** 2025-11-24

**Authors:** Jintapa Sueasuay, Chawikan Boonwong, Rawipas Saisuwan, Nuttawan Kassaket, Issara Prachongsai, Wiwit Tantibhedhyangkul, Patimaporn Wongprompitak, Yupin Suputtamongkol, Pattama Ekpo, Naharuthai Inthasin

**Affiliations:** 1 Department of Immunology, Faculty of Medicine Siriraj Hospital, Mahidol University 65106, Bangkok, Thailand; 2 Research Department, Faculty of Medicine Siriraj Hospital, Mahidol University 65106, Bangkok, Thailand; 3 Department of Medical Technology, School of Allied Health Sciences, Walailak University 65133, Nakhon Si Thammarat, Thailand; 4 Department of Medicine, Faculty of Medicine Siriraj Hospital, Mahidol University 65106, Bangkok, Thailand; Icahn School of Medicine at Mount Sinai, New York, New York, USA

**Keywords:** leptospirosis, acute phase, diagnosis, immunochromatographic test, immunofluorescent assay, rapid test

## Abstract

We have computationally identified a conserved region of flagellin B (FlaB) protein that is present only in pathogenic *Leptospira* but absent in non-pathogenic *Leptospira* or other flagellated bacteria. The predicted FlaB subunit protein (sFlaB) comprised a sequence of 50-amino acids: ^140^FARGSRVASMWFHMGPNQNQRERFYIGTMTSKALKLVKADGRPIAISSPG^189^. The nucleotide sequence encoding sFlaB was amplified by polymerase chain reaction from *Leptospira* genomic DNA and cloned into a pET100 expression vector. The recombinant sFlaB was expressed in *Escherichia coli* BL21 and affinity purified to serve as an antigen to the immunochromatographic test (ICT) designed for specific IgM (ICT-IgM) and IgG (ICT-IgG) antibody detections. We evaluated 109 serum samples, including 46 from leptospirosis patients (29 acute sera and 17 convalescent sera) and 63 from patients with other acute febrile illnesses (46 acute sera and 17 convalescent sera). Compared to the paired serum results of the indirect immunofluorescent assay, the sensitivity of ICT-IgM and ICT-IgG was 80.40% and 71.73%, with specificities of 84.13% and 73.01%, respectively. For acute leptospirosis sera, ICT-IgM showed a sensitivity of 75.86% and a specificity of 89.13%, while ICT-IgG had a sensitivity of 68.97% and a specificity of 71.94%.

**IMPORTANCE:**

Leptospirosis is a zoonotic disease caused by pathogenic leptospires. The infected patient presents with a mild to severe febrile illness and may die while receiving inappropriate treatment. The microscopic agglutination test, the current gold standard method, is laborious and requires the use of live panel leptospires, which should only be done in a reference laboratory. In addition, the results of the paired serum samples are required for an accurate interpretation. Polymerase chain reaction (PCR) was used instead for diagnosis in the acute phase of infection. However, PCR requires an expensive machine and a specialist to analyze the results. Therefore, a simple and rapid test is needed for the early diagnosis of leptospirosis.

## INTRODUCTION

Leptospirosis is a zoonotic infection caused by pathogenic spirochetes of the genus *Leptospira*. This reemerging disease is a common cause of febrile illness in tropical regions worldwide (1). The symptoms of leptospirosis are highly variable and can mimic other febrile illnesses, such as dengue fever, malaria, melioidosis, and scrub typhus (2). A prompt diagnosis is critical for effective antibiotic therapy, as the treatment is most beneficial when initiated early (3). The gold standard laboratory methods are culture isolation and the microscopic agglutination test (MAT) (4, 5). However, these methods are limited by their low sensitivity, poor reproducibility, and time-consuming nature (6–10). In addition, MAT requires a panel of live leptospiral antigens that are specific to a geographic area (11–13). Therefore, these tests are only available in specialized reference laboratories. To address these limitations, rapid serological tests such as the IgM-enzyme-linked immunosorbent assay (ELISA) and the lateral flow assay have been developed. Most commercial IgM assays utilize whole-cell lysates or crude outer membrane components such as lipopolysaccharide and outer membrane proteins (10, 13–16), which are prone to cross-reactivity and reduced specificity in the co-endemic regions.

Recombinant antigens are widely used due to the difficulties associated with leptospiral cultivation, which is time-consuming, risks infection, and requires multiple steps during antigen preparation. Recombinant proteins offer advantages such as large-scale production, high recovery rate, and high antigen purity. Various recombinant antigens, including flagellin, heat shock protein, outer membrane protein (e.g., LipL21, LipL22, LipL32, LipL41, LigA, LigB, OmpL1), and leptospiral surface adhesion proteins have been used for diagnostic purposes (17–24). However, a low sensitivity during the early stage of the disease and cross-reactivity with other febrile illnesses are still being challenged.

*Leptospira* spp. possess two periplasmic flagella composed primarily of the structural proteins flagellin A (FlaA) and flagellin B (FlaB), with FlaB forming the filament core (25, 26). Like other bacterial flagellins, FlaB consists of four structural domains (D0–D3) (27–31), where the conserved D0 and D1 domains, located at the N and C termini, are known to activate innate immune responses via pattern recognition receptors such as TLR5 and NAIP inflammasomes (27, 28, 31–34). In contrast, the central domains (D2 and D3) are typically variable and immunogenic, eliciting humoral responses (28, 31). Interestingly, *Leptospira* FlaB lacks the typical D2 and D3 domains but retains a short hypervariable sequence in the central region (35, 36). This region is conserved among pathogenic *Leptospira* species but is less homologous to proteins from non*-Leptospira* pathogens and unrelated flagellated bacteria. Therefore, it represents a promising antigenic candidate for the development of a rapid diagnostic test.

Although FlaB is not a surface-exposed antigen, its selection was based on its immunological relevance. TLR5 is known to recognize bacterial flagellin in its monomeric form, which is released upon bacterial degradation rather than from intact flagellar filaments (32). In *Leptospira*, FlaB has been shown to activate human TLR5, especially following membrane disruption by host antimicrobial peptides, as demonstrated by Holzapfel et al. (36). This activation implies that FlaB is accessible to the immune system during infection and can contribute to both innate and adaptive immune responses. Furthermore, proteomic analyses using 2D PAGE, immunoblotting, and MALDI-TOF MS revealed high expression levels of FlaB in more virulent *Leptospira* serovars. Immunoblots probed with patient IgM and IgG sera confirmed the immunogenicity of FlaB, as the protein spot corresponding to FlaB showed strong reactivity, particularly in correlation with high MAT titers (37, 38). These findings support FlaB as a relevant immunogen despite its non-surface localization. It has been shown to be immunodominant with robust, strong anti-FlaB responses observed in both patient and animal sera (37). Evidence from a previous study using 2D-gel electrophoresis revealed the dominant presence of anti-FlaB antibody in the sera of leptospirosis patients and infected mice (37, 39). Furthermore, higher levels of blood expression of the FlaB gene have been reported in susceptible hamsters compared to resistant mice (40). While full-length FlaB has been used successfully in bovine serodiagnostics (17), its application in human diagnostics remains limited, partly due to concerns about cross-reactivity from its conserved termini. A monoclonal antibody (M138) specific to FlaB further supports its antigenic potential, particularly in the variable central region of *Leptospira* serovar Pomona (35), which remains underexplored for diagnostic development.

While full-length FlaB is known to be immunogenic, it includes conserved N-terminal and C-terminal domains that can result in cross-reactivity with antibodies from other spirochetal or flagellated bacterial infections. To improve diagnostic specificity, we computationally analyzed flagellin sequences and identified a 50-amino acid segment from the central variable region of FlaB, called sFlaB. The sFlaB was selected based on its strong B-cell reactivity and reduced sequence homology with other spirochete and non-pathogenic *Leptospira* species. By excluding conserved domains, sFlaB aims to minimize non-specific antibody binding and enhance diagnostic specificity—an important consideration in co-endemic settings where cross-reactivity and false positives are a major concern.

In this study, recombinant sFlaB was expressed, purified, and employed in immunochromatographic tests (ICTs) for IgM and IgG detection. Compared to indirect immunofluorescent assay (IFA) using paired sera, ICT-IgM demonstrated potential for early-phase screening of leptospirosis from single serum samples.

## MATERIALS AND METHODS

### Selection of a specific region of FlaB protein

The FASTA format of amino acid sequences of bacterial flagellin proteins was obtained from the NCBI database (Table 1). The sequences were aligned and compared using the constraint-based multiple alignment tool (NCBI) and CLUSTAL Omega (EMBL-EBI). The B-cell epitope prediction was computationally identified by BepiPred 3.0 (Department of Health Technology, Lyngby, Denmark) (41, 42).

**TABLE 1:** GenBank accession number of the FASTA sequence from NCBI database

Bacteria		GenBank accession number
Pathogenic *Leptospira* spp.	*Leptospira interrogans*	WP_000586171.1 flagellin
	*Leptospira kirschneri*	WP_004777615.1 flagellin
	*Leptospira weilii*	WP_061231082.1 flagellin
	*Leptospira borgpetersenii*	WP_194491554.1 flagellin
	*Leptospira kmetyi*	WP_010574971.1 flagellin
	*Leptospira alstonii*	WP_020772736.1 flagellin
	*Leptospira alexanderi*	BAP75409.1 flagellin, partial
Non-pathogenic *Leptospira* spp.	*L. yanagawae*	TGL19238.1 flagellin
	*Leptospira idonii*	WP_135761406.1 flagellin
	*L. vanthielii*	TGM51636.1 flagellin
	*Leptospira meyeri*	WP_004789111.1 flagellin
	*Leptospira terpstrae*	WP_039937888.1 flagellin
	*Leptospira biflexa*	WP_012476288.1 flagellin
Other flagellated bacteria	*B. burgdorferi*	WP_106014331.1 flagellin FlaB
	*B. subtilis*	SNY60529.1 flagellin
	*Salmonella enterica subsp. Enterica serovar Typhi*	PJM45236.1 flagellin FliC
	*Treponema pallidum*	WP_271425553.1 flagellin
	*Pseudomonas aeruginosa*	OHP43595.1 flagellin
	*H. pylori*	AAA25016.1 flagellin
	*Escherichia coli*	KIH03896.1 flagellin

### *Leptospira* cultivation and DNA extraction

*L. interrogans* serovar Autumnalis was isolated from a clinical specimen obtained from a patient admitted to Siriraj Hospital, Bangkok, Thailand. The bacteria were cultured in Ellinghausen-McCullough-Johnson-Harris medium, supplemented with 10% of leptospiral enrichment (HiMedia Laboratories, Mumbai, India) at 30°C. After cultivation, the bacteria were harvested by centrifugation at 10,000 × *g* for 10 min. The total DNA from *Leptospira* was extracted by using a DNA extraction kit (Qiagen, Hilden, Germany) following the manufacturing protocol.

### Cloning, expression, and purification of conserved pathogenic *Leptospira* flagellin B subunit protein (conserved FlaB subunit)

The forward primer 5′-CACCATGTTCGCTAGAGGTTCCAGGGT-3′ and reverse primer 5′-TTATCCCGGAGAAGAGATCGC-3′ were used to amplify the leptospiral *flaB* gene encoding 50-amino acids of *L. interrogans sFlaB*. The polymerase chain reaction (PCR) includes denaturation at 94°C for 30 s, annealing at 55°C for 30 s, and extension at 72°C for 1 min. The 5′ end of the amplified PCR product was ligated into the GTGG overhang in the pET100 directional TOPO expression vector under the T7 promoter (Invitrogen, Carlsbad, CA, USA). The recombinant plasmid was propagated and maintained in the One Shot TOP10 *Escherichia coli* (Invitrogen) before being transformed and expressed in the BL21 Star (DE3) *E. coli* strain (InvivoGen). The transformed *E. coli* was cultured in Luria-Bertani broth (BD, MA, USA) at 37°C until OD_600_ = 0.4. Then, 0.5 mM isopropyl β-D-1-thiogalactopyranoside (IPTG) was added and further incubated for 4–6 h. The *E. coli* pellet was harvested and sonicated to collect the *E. coli* supernatant. The expressed protein was purified by Probond Nickel-Chelating Resin (Invitrogen, CA, USA) following the manufacturing protocol. The purity of protein was analyzed by SDS-PAGE and Western blotting.

### Serum samples

The study was conducted prospectively in a hospital-based setting, enrolling patients clinically suspected of leptospirosis according to the Thai Ministry of Public Health diagnostic guidelines (2021). The 109 serum samples were kindly supported by Prof. Yupin Suputthamongkol, Department of Medicine, Faculty of Medicine Siriraj Hospital. The use of patient serum was approved by the Siriraj Institutional Review Board (COA no. Si014/2019), Faculty of Medicine Siriraj Hospital, Mahidol University. Among 109 serum samples, 46 were from leptospirosis patients (29 acute sera and 17 convalescent sera), while the remaining 63 were from patients with unidentified febrile illness (UFI) (46 acute sera and 17 convalescent sera). Acute sera were collected on the first date the patient arrived at the hospital. The convalescent serum was then collected 1–2 weeks later (11 ± 6 days). These sera are diagnosed by clinical manifestation and IFA. The IFA had been evaluated and applied for routine service for the diagnosis of leptospirosis at Siriraj Hospital, Thailand (43). The IFA is certified under ISO 15189. The fourfold increase in Ab titers between paired sera confirmed leptospirosis. For other acute febrile illness sera, *Leptospira* IgM/IgG-Ab in the IFA titer was less than 1:50 in both acute and convalescent sera. Pooled serum samples from patients with confirmed scrub typhus or dengue infection were used during assay optimization. Scrub typhus was diagnosed by a fourfold increase in IgG or IgM titers against *Orientia tsutsugamushi* serogroups (Karp, Gilliam, and Kato) between paired acute and convalescent sera, as determined by IFA. Dengue hemorrhagic fever (DHF) was confirmed by NS1 antigen detection and ELISA for NS1-specific IgM and IgG antibodies. All assays were performed in laboratories accredited under ISO 15189 standards.

### Immunofluorescent assay

The IFA protocol followed the method described by Appassakij et al. (44). Briefly, whole-cell *Leptospira* were smeared onto clean glass slides, air-dried, and fixed with acetone at 4°C for 10 min. The slides were stored at −70°C until use. Patient sera were heat-inactivated and serially diluted in PBS at ratios of 1:50, 1:100, 1:200, 1:400, 1:800, 1:1,600, 1:3,200, and 1:6,400. Five microliters of each diluted serum sample was applied to the antigen spots and incubated in a moist chamber at 37°C for 30 min. After washing with PBS, 5 µL of FITC-conjugated rabbit anti-human IgG or IgM (Dako, Glostrup, Denmark) was added and incubated at 37°C for an additional 30 min. The slides were then washed, air-dried, mounted in buffered glycerol, and examined under a fluorescence microscope equipped with FITC-specific filters at 400× magnification (Bx50, Olympus Optical Co., Ltd., Tokyo, Japan). *Leptospira* exhibiting apple-green fluorescence with typical spirochete morphology were considered positive. Positive and negative control sera were included in each run. Endpoint titers were determined as the highest serum dilution showing specific fluorescence.

### Assembly of an immunochromatographic test strip

**Fig 1:**
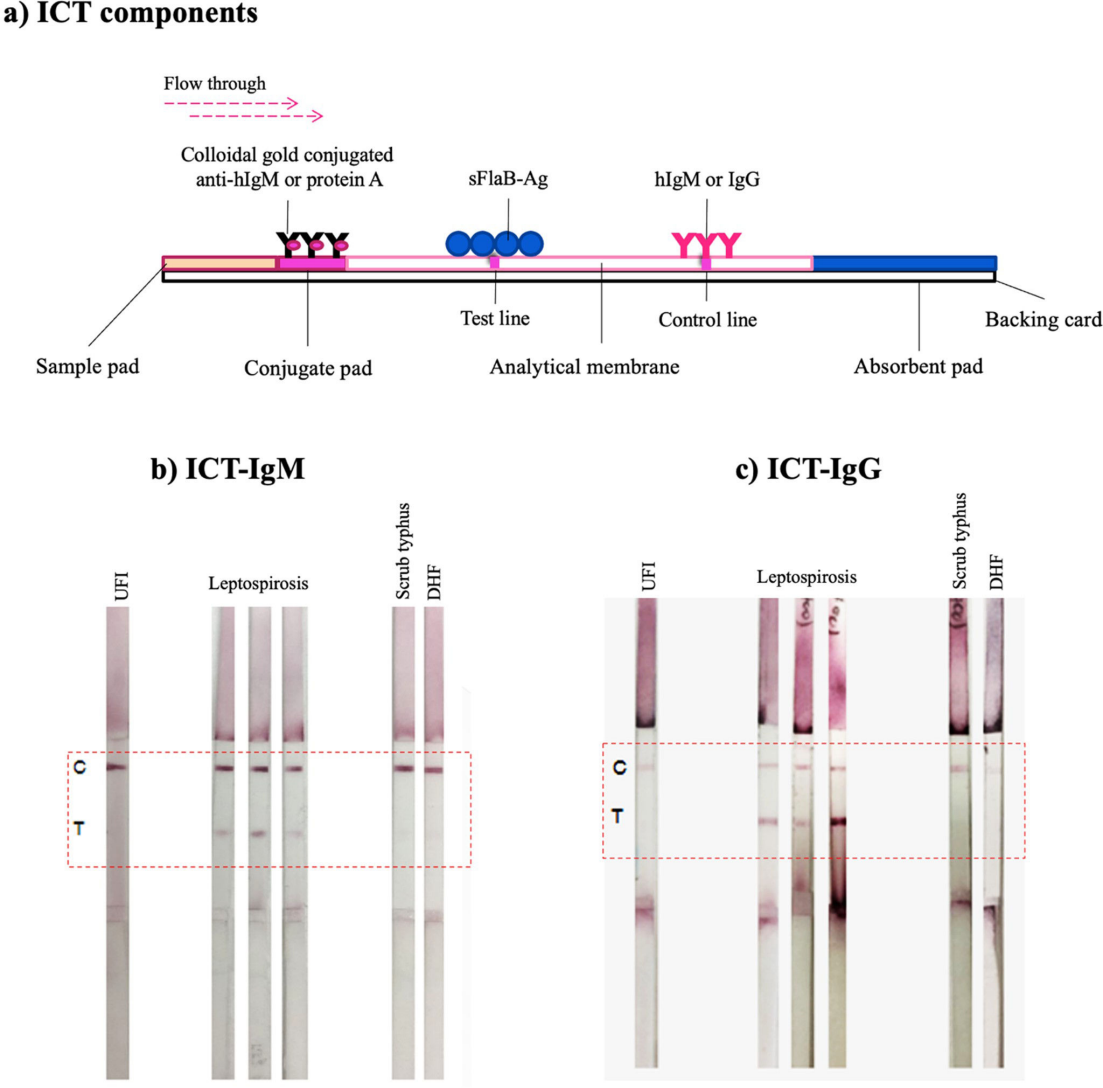
(**a**) Schematic illustration of the ICT device, with labeled components including the sample pad, conjugate pad, analytical membrane (featuring test and control lines), and absorbent pad. ICT results for IgM (**b**) and IgG (**c**) tests using pooled serum samples from patients with confirmed leptospirosis, UFI, scrub typhus, and DHF.

ICTs were developed to detect specific antibodies (Abs). The strip assembly consists of a sample pad (CF3, Whatman International Ltd., Maidstone, UK), conjugate pad, analytical membrane (Prima85, Whatman), and absorbent pad (CF3, Whatman). The conjugated pad was soaked in 0.1 M potassium phosphate buffer pH 7.4 (PBS) containing 0.5% BSA, 0.1% Tween-20 for 5 min and then dried at 37°C for 30 min. The colloidal gold-conjugated protein A or anti-human IgM-Ab (Pacific Biotech, Phetchabun Province, Thailand), containing 2% trehalose, 10% sucrose, was sprayed onto a conjugate pad at a dispensing rate of 10 µL/cm. The pad was then dried at 37°C for 1 h. The recombinant sFlaB (1 mg/mL in 1% BSA, 0.01% Tween-20/PBS) was applied to a nitrocellulose membrane at the test band (T) position of the strip. Human (h)IgG or hIgM (1 mg/mL in 1% BSA, 0.1% Tween-20/PBS) (Sigma-Aldrich, Darmstadt, Germany) was applied to the same nitrocellulose membrane in a position of the control band (C). The membrane was blocked with 1% BSA, 0.1% Tween-20/PBS. After being washed two times with 0.1% Tween-20/PBS and deionized water, the membrane was dried at 37°C for 20 min. The sample pad, conjugate pad, analytical membrane, and absorption pad were assembled on the backing card. The assembled card was cut and stored in a plastic bag inside a desiccator. The ICT assembly is shown in Fig. 1a.

### Immunochromatographic test strip protocol

During the initial optimization phase, we employed pooled serum samples from confirmed *Leptospira*-infected patients as positive controls, along with pooled sera from patients with undifferentiated febrile illnesses as negative controls. Additionally, we included samples from patients with confirmed dengue and scrub typhus infections to assess potential cross-reactivity. Using serial dilution testing, we evaluated the performance of the sFlaB-ICT across a range of dilutions. For IgM detection, the 1:200 dilution consistently produced strong signal intensity and clear discrimination between *Leptospira*-positive samples and the negative and non-*Leptospira* controls (Fig. 1b). For IgG detection, a 1:1,200 dilution provided the best contrast, minimizing background noise while maintaining sufficient sensitivity to detect true positives (Fig. 1c). These dilution choices were therefore guided by empirical results to maximize both the sensitivity and specificity of the ICT assay in differentiating true positive from negative and cross-reactive samples.

The serum was diluted to 1:200 and 1:1,200 dilutions in an application buffer (0.1% Tween-PBS) for specific IgM and IgG detections, respectively. Fifty microliters of the diluted serum was applied to the sample pad, followed by 100 µL of application buffer. The serum migrated to the conjugate pad, the specific Abs in the serum reacted with microparticles in the conjugate, and then continued to the analytical membrane. A red-purple band will appear at the T location within 15 min. If there are no specific Abs, no band will appear. The control band at the C location will always be visible, confirming that the test has been performed correctly.

## RESULTS

### Selection of the conserved specific amino acid sequence of the pathogenic *Leptospira* FlaB protein (sFlaB)

**Fig 2:**
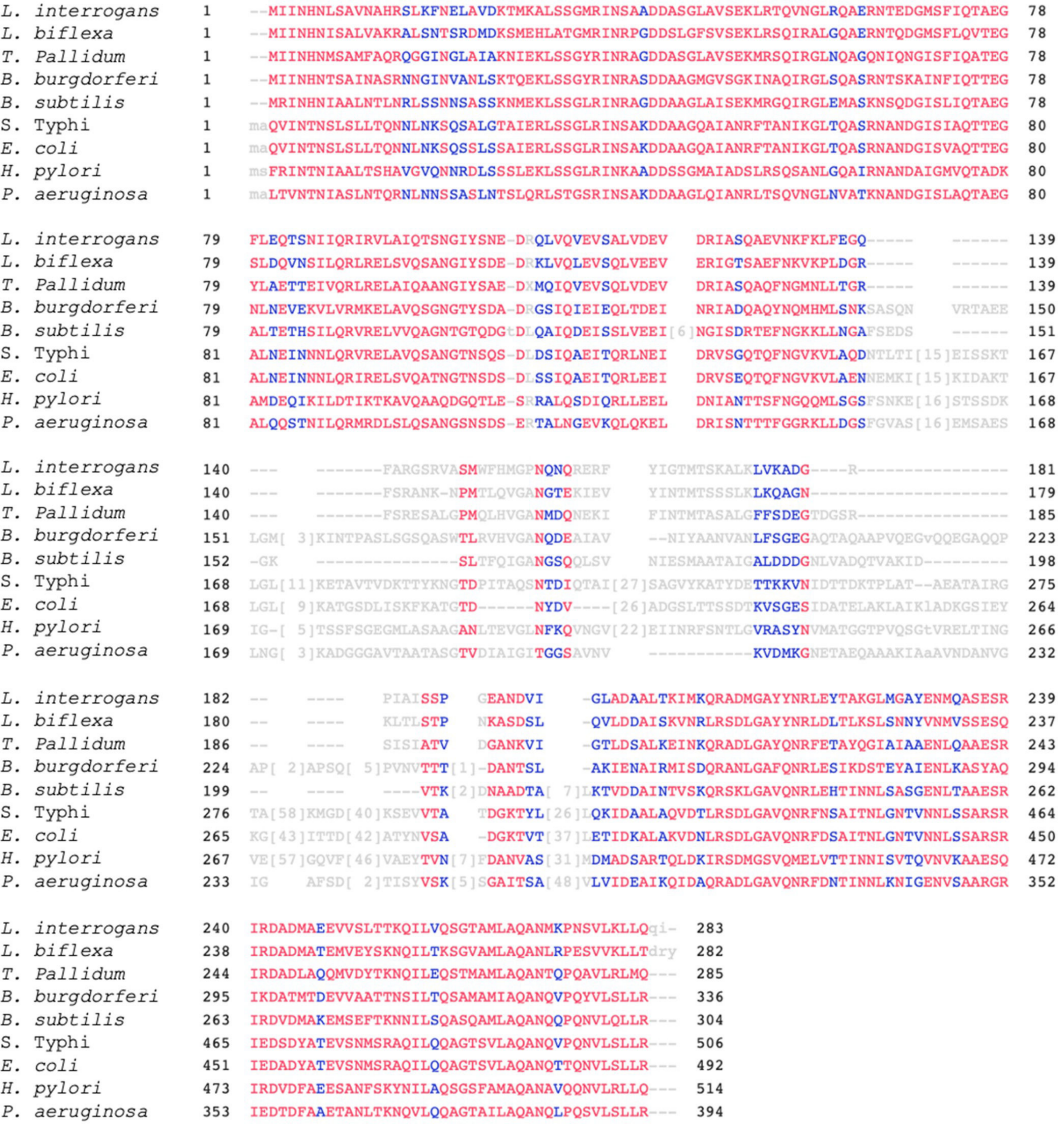
Multiple sequence alignment of bacterial flagellin proteins using the constraint-based multiple alignment tool from NCBI and CLUSTAW Omega (EMBL-EBI). The alignment showed conservation of amino acids at the N-terminal region (1-139) and the C-terminal region (190-283), while the central region is highly variable between different bacterial species. Red font indicates highly conserved positions, and blue font indicates lower conservation.

**Fig 3:**
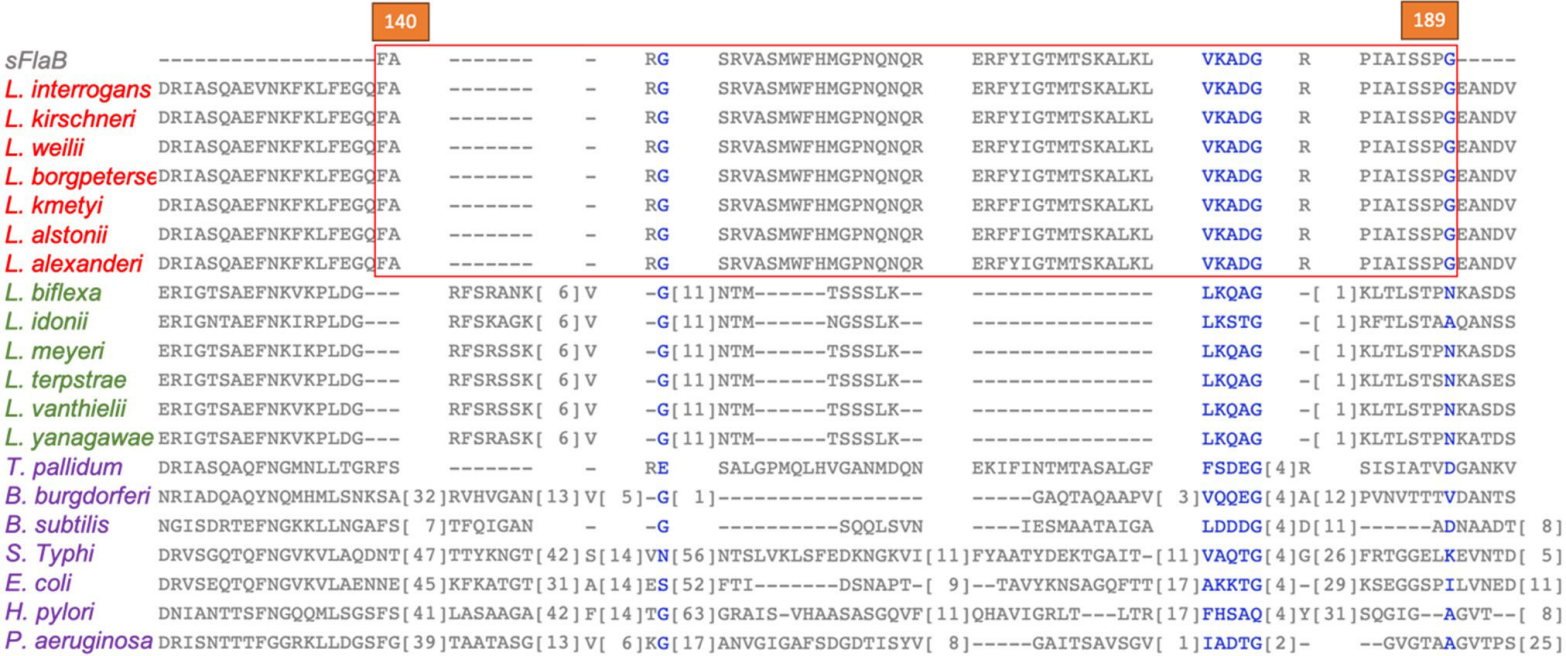
Amino acid sequence at positions 140-189 of FlaB (sFlaB) was compared with those of pathogenic *Leptospira* (red alphabets), non-pathogenic *Leptospira* (green alphabets), and other flagellated bacteria (purple alphabets).

**Fig 4:**
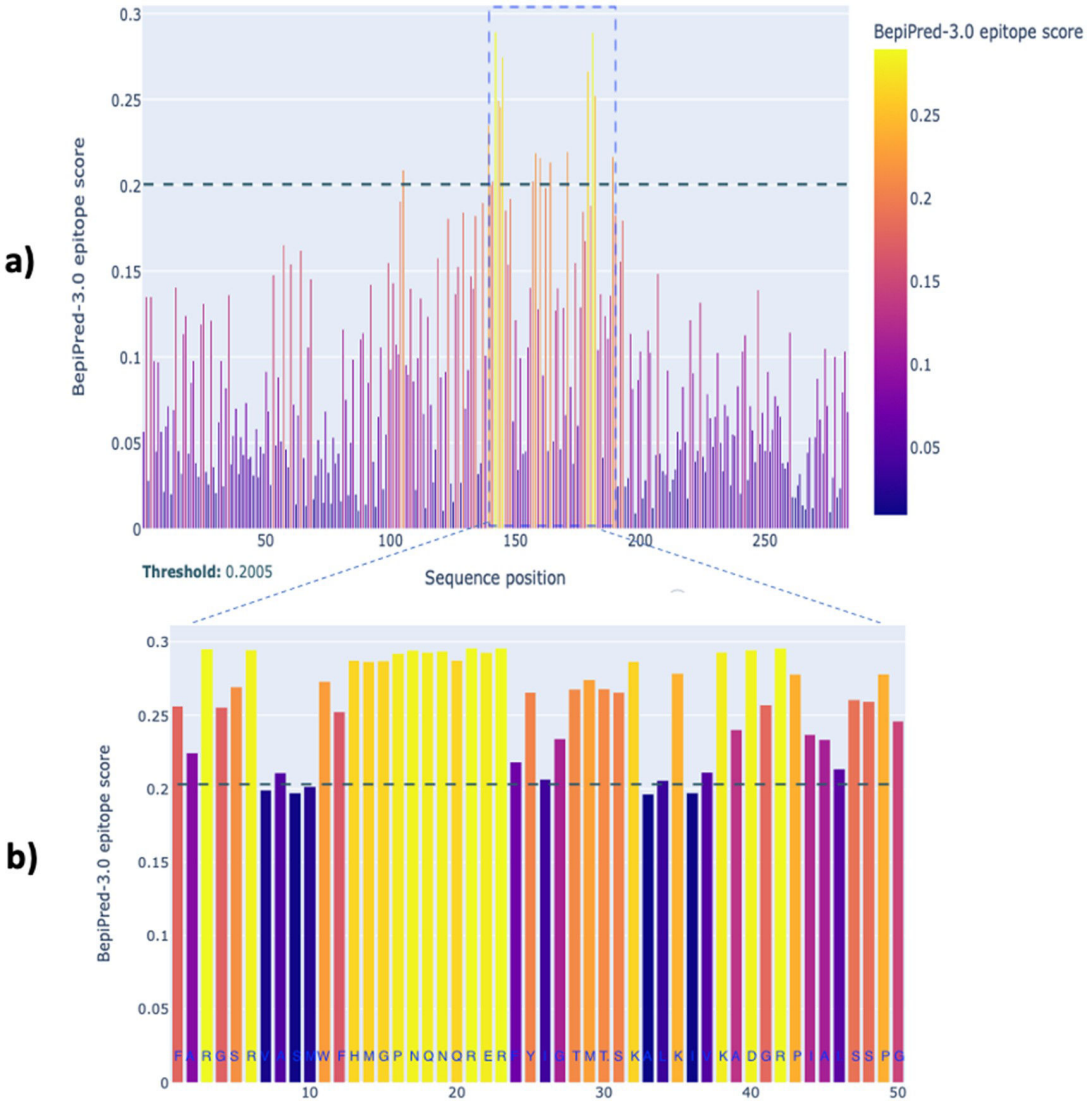
B-cell epitope prediction of *L. interrogans* whole flagellin (**a**) and sFlaB protein (**b**). The amino acid sequence of *L. interrogans* flagellin (WP000586171.1) taken from the NCBI database was analyzed by BepiPred-3.0. The result revealed a high B-cell epitope score predominantly within sFlaB (cropped in panel **a** and amplified in panel **b**). The *X*-axis represents the amino acid position, and the *Y*-axis represents an epitope score.

To identify the conserved sequence, the amino acid sequences of the bacterial flagellin proteins taken from the NCBI database (Table 1) were aligned using both the constraint-based multiple alignment tool (NCBI) and CLUSTAW Omega (EMBL-EBI). Similar to other flagellated bacteria, *Leptospira* FlaB protein exhibits conserved amino acids at the N and C termini at amino acid positions 1-139 and 190-283, respectively (Fig. 2 and 3). Fifty amino acids in a central region, ^140^FARGSRVASMWFHMGPNQNQRERFYIGTMTSKALK LVKADGRPIAISSPG^189^ (sFlaB), showed variability between various flagellated bacteria (*Salmonella* Typhi, *Escherichia coli*, *Pseudomonas aeruginosa*, *Bacillus subtilis*, *Treponema pallidum*, *Helicobacter pylori*, and *Borrelia burgdorferi*), as well as non-pathogenic *Leptospira* (*L. biflexa*, *L. vanthielii*, *L. yanagawae*, *L. idonii*, *L. meyeri*, and *L. terpstrae*). In contrast, sFlaB was conserved among pathogenic *Leptospira* strains (*L. interrogans*, *L. kirschneri*, *L. weilii*, *L. borgpetersenii*, *L. kmetyi*, *L. alstonii*, and *L. alexanderi*) (Fig. 2 and 3). A BLAST search of the NCBI protein database confirmed that the sFlaB is 100% identical to that found in pathogenic *Leptospira*, but is absent in other bacterial species. To further evaluate the potential of sFlaB as an antigenic determinant, we used BepiPred 3.0 to predict B-cell epitopes. The result indicated that the region with a high epitope score is predominantly located within amino acids 140-189 (Fig. 4). Based on these findings, sFlaB was constructed and incorporated into the development of an ICT kit for specific antibody detection.

### Production of the recombinant sFlaB protein

**Fig 5:**
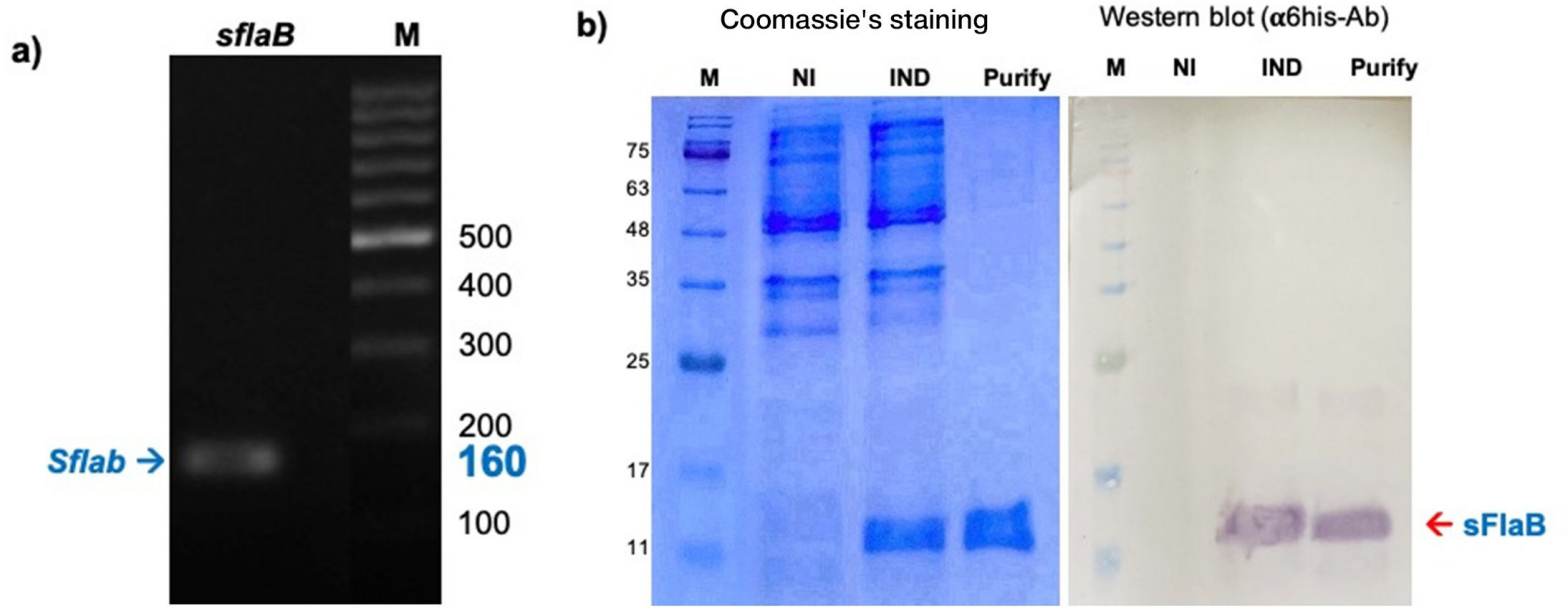
Cloning, expression, and purification of recombinant sFlaB protein. The gene encoding sFlaB protein was amplified by PCR, generating the 160 bp product (**a**). The *flaB* gene was then inserted into the pET100 directional TOPO expression vector that generates recombinant plasmid to express in BL21 Star (DE3) *E. coli*. The affinity-purified expressed sFlaB protein was shown on 15% SDS-PAGE staining with Coomassie’s blue and Western blotting reacted with anti-6-His (**b**). M, molecular weight marker; NI, non-IPTG induction; IND, IPTG induction.

To construct a recombinant sFlaB protein, the gene encoding the 50-amino acid sequence of sFlaB was amplified from the *L. interrogans* DNA template, resulting in a 160 bp PCR product (Fig. 5a). This PCR product was then ligated into the pET100 TOPO vector and transformed into *E. coli*. Following induction with IPTG, a 10 kDa 6-His-tagged sFlaB recombinant protein was expressed and subsequently purified using affinity chromatography. The successful expression of the recombinant sFlaB protein was confirmed by 15% SDS-PAGE and Western blotting using an anti-6-His-Ab (Fig. 5b).

### Evaluation of the sensitivity and specificity of ICT tests based on IFA paired serum analysis results

For acute serum samples, ICT-IgM and ICT-IgG were evaluated using sera from 29 leptospirosis patients and 46 patients with other febrile illnesses. The ICT-IgM exhibited a sensitivity of 75.86% and a specificity of 89.13%. In comparison, the ICT-IgG assay demonstrated a sensitivity of 68.97% and a specificity of 71.74% (Table 2). When combining the results of both ICT-IgM and ICT-IgG assays, the sensitivity increased to 89.66%, although the specificity decreased to 65.22% (Table 3).

**TABLE 2:** Evaluation of sensitivity, specificity, NPV, PPV of ICT-IgM, and ICT-IgG kits in acute and convalescent sera compared with IFA results

Evaluation	Acute sera		Convalescent sera		All serum samples	
	ICT-IgM	ICT-IgG	ICT-IgM	ICT-IgG	ICT-IgM	ICT-IgG
% Sensitivity	75.86 (22/29)	68.97 (20/29)	88.24 (15/17)	76.47 (13/17)	80.43 (37/46)	71.73 (33/46)
% Specificity	89.13 (41/46)	71.74 (33/46)	70.59 (12/17)	76.47 (13/17)	84.13 (53/63)	73.01 (46/63)
% PPV	81.48 (22/27)	60.61 (20/33)	75.00 (15/20)	76.47 (13/17)	78.72 (37/47)	66.00 (33/50)
% NPV	85.42 (41/48)	78.57 (33/42)	85.71 (12/14)	76.47 (13/17)	85.48 (53/62)	77.97 (46/59)

**TABLE 3:** Evaluation of the sensitivity, specificity, PPV, NPV of combined ICT-IgM and ICT-IgG results compared with IFA results

Evaluation	Acute sera	Convalescent sera	All serum samples
	ICT-(IgG + IgM)	ICT-(IgG + IgM)	ICT-(IgG + IgM)
% Sensitivity	89.66 (26/29)	100 (17/17)	93.48 (43/46)
% Specificity	65.22 (30/46)	47.06 (8/17)	60.32 (38/63)
% PPV	61.90 (26/42)	65.38 (17/26)	63.32 (43/68)
% NPV	90.91 (30/33)	100 (8/8)	92.68 (38/41)

For convalescent serum samples, the ICT-IgM and ICT-IgG assays were evaluated using sera from 17 leptospirosis patients and 17 patients with other febrile illnesses. The ICT-IgM showed a sensitivity of 88.24% and a specificity of 70.59%. In comparison, the ICT-IgG showed both a sensitivity and a specificity of 76.47% (Table 2). Combining the results of both ICT-IgM and ICT-IgG assays achieved 100% sensitivity, though the specificity was reduced to 47.06% (Table 3).

For the overall sample set, including acute and convalescent serum samples, the ICT-IgG assay had a sensitivity of 71.73% and a specificity of 73.01%. The ICT-IgM assay showed a sensitivity of 80.43% and a specificity of 84.13% (Table 2). Combining the results of the ICT-IgM and ICT-IgG assays yielded a sensitivity of 93.48% and a specificity of 60.32% (Table 3).

Together, the ICT-IgM assay demonstrates higher sensitivity and specificity compared to the ICT-IgG assay, especially in acute serum. However, for convalescent serum samples, the specificity of both tests is similar. Combining the results of the ICT-IgM and ICT-IgG assays enhances the sensitivity to between 90% and 100%, although this combination significantly reduces specificity. Given its superior performance, the ICT-IgM assay is more effective in the screening of leptospira infections compared to ICT-IgG. Consequently, we have focused on evaluating ICT-IgM with single acute serum samples for improved diagnostic accuracy.

### ICT-IgM had higher sensitivity than IFA-IgM and IFA-IgG in the evaluation of a single acute serum sample evaluation

Among 29 acute leptospirosis sera confirmed by a fourfold rise in antibody titers in paired sera using IFA and clinical manifestation, only 17 of 29 samples exhibited IFA antibody titers greater than 1:200 (Table 4). With a cutoff of 1:200 for the single serum interpretation, the sensitivity of IFA-IgM, IFA-IgG, and both IFA-IgM + IFA-IgG was only 58.62% (17/29), 17.24% (5/29), and 58.62% (17/29), respectively. In contrast, our ICT-IgM assay identified 22 of 29 patients as positive, demonstrating a sensitivity of 75.86% (Table 4). These results suggest that our ICT-IgM assay is more sensitive than IFA-IgM and IFA-IgG for the evaluation of a single acute serum sample. Furthermore, for acute sera from patients with unidentified febrile illnesses, ICT-IgM showed a false positive rate of 10.87% (5/46), corresponding to a specificity of 89.13%. Therefore, our ICT-IgM assay offers a high sensitivity and specificity, compared to IFA when evaluated with single acute serum samples.

**TABLE 4:** The results of ICT and IFA tests with the 29 confirmed acute leptospirosis sera

No.	IFA		ICT	
	IgM	IgG	IgM	IgG
1	1:1,600	<1:50	Pos	Neg
2	<1:50	<1:50	Pos	Neg
3	<1:50	1:100	Pos	Pos
4	1:200	<1:50	Pos	Pos
5	1:3,200	1:3,200	Pos	Pos
6	<1:50	<1:50	Pos	Pos
7	<1:50	<1:50	Neg	Pos
8	1:400	<1:50	Neg	Neg
9	1:3,200	<1:50	Pos	Pos
10	1:200	1:50	Pos	Neg
11	<1:50	1:100	Neg	Pos
12	≥1:6,400	1:200	Pos	Pos
13	≥1:6,400	1:50	Pos	Pos
14	1:50	<1:50	Pos	Neg
15	1:800	1:100	Neg	Neg
16	<1:50	<1:50	Pos	Neg
17	<1:50	<1:50	Pos	Pos
18	1:3,200	1:3,200	Pos	Pos
19	<1:50	<1:50	Pos	Pos
20	<1:50	<1:50	Pos	Pos
21	1:400	<1:50	Neg	Neg
22	1:3,200	<1:50	Pos	Pos
23	1:200	1:50	Pos	Pos
24	<1:50	1:100	Neg	Pos
25	≥1:6,400	1:200	Pos	Pos
26	1:800	<1:50	Pos	Pos
27	1:400	1:1,600	Neg	Pos
28	≥1:6,400	1:50	Pos	Pos
29	1:50	<1:50	Pos	Neg

## DISCUSSION

The MAT is a gold standard for laboratory diagnosis of leptospirosis. However, the test is typically limited to reference laboratories due to its complexity. An alternative method, IFA, is commonly employed in large hospitals (43) and is routinely available at Siriraj Hospital in Thailand (certified under ISO 15189). Several studies have demonstrated that IFA offers diagnostic performance comparable to the MAT (42), particularly during the acute phase of infection. For instance, the study conducted in Songkhla, Thailand, found that IFA had the highest sensitivity (91.9%) and excellent specificity (100%) among the serological methods evaluated, whereas MAT showed a lower sensitivity of 76.6%, despite also maintaining 100% specificity (45). These findings support the use of IFA as a suitable and reliable tool for the routine clinical diagnosis of leptospirosis. However, the limitation of IFA is a multistep procedure and requires specialized personnel and equipment (13, 45). Therefore, it is less suitable for rapid screening tests or use in rural or field settings.

Early diagnosis is crucial for effective antibiotic treatment, highlighting the need for a simple and rapid screening method for leptospirosis, particularly during the acute phase of symptoms. ICT kit is widely used in various serological tests because it is rapid, simple, easy to perform outside the laboratory, and does not require specialized equipment or expert personnel. Therefore, ICT is suitable to screen for leptospirosis. Confirmatory laboratory tests, such as MAT or paired serum test, using other serological methods such as IFA, can be performed later.

In this study, we developed ICT for serodiagnostic screening of leptospirosis using a newly constructed *Leptospira* flagellin B, sFlaB, protein as the antigen. The flagellin protein has been used in the laboratory diagnosis of various infectious diseases. For example, the flagellin protein of *S. Typhimurium* (aa 171-303) has been used to develop an IgM-ELISA in serological testing of typhoid fever. The sensitivity, specificity, positive, and negative predictive values of the test were 83.7%, 99.0%, 97.5% and 92.8%, respectively (46). Previous studies have shown that the full-length 35 kDa FlaB protein exhibits antigenicity, reacting with anti-*Leptospira* IgM and IgG in human leptospirosis sera (35, 37). Additionally, it has been used in a fluorescent polarization assay to detect anti-*Leptospira* antibodies in bovine but not in human sera, achieving positive and negative predictive values of 81.7% and 83.3%, respectively (17). However, a unique subunit of pathogenic specific FlaB antigen located in the central variable domains will be more suitable than using the whole FlaB. The whole FlaB contains conserved amino acids at both the N and C termini, which results in cross-reactivity with other infectious diseases caused by flagellated bacteria. Therefore, we analyzed, selected, and constructed the 50-amino acid from the central region of *L. interrogans* flagellin B (^140^FARGSRVASMWFHMGPNQNQRERFYIGTMTSKALKLVKADGRPIAISS PG^189^), which is conserved among pathogenic *Leptospira* strains and contains B-cell epitopes involved in antibody responses. The performance of our developed ICT was evaluated using 109 sera (46 leptospirosis cases and 63 from patients with other febrile illnesses), confirmed by a fourfold rise in Ab titer in paired sera by IFA and clinical manifestation (Tables 2 and 3). Compared to IFA, our developed ICT-IgM demonstrated a sensitivity of 80.43%, specificity of 84.13%, PPV of 78.72%, and NPV of 85.48%. In contrast, ICT-IgG showed a sensitivity of 71.73%, a specificity of 73.01% with 66.00% PPV and 77.97% NPV. Our developed ICT-IgM has higher sensitivity and specificity than ICT-IgG. Combining the ICT-IgM and ICT-IgG results can enhance sensitivity to 90%–100%, but the specificity is relatively low.

For acute sera, the sensitivity of serological tests is typically low. For example, the sensitivity of MAT is about 41% during the first week of the illness (47). Although commercial IgM-ELISAs and IgM dipstick tests are widely used, their diagnostic performance during the acute phase varies significantly. Reported sensitivities range from 1.8% to 75% and specificities from 36.4% to 97.7%, depending on the test kit and study population (46, 48–50). These limitations hinder early detection, particularly during the acute phase of illness. In our study, we evaluated 75 acute sera (29 leptospirosis and 46 other febrile illnesses). The IFA demonstrated a sensitivity of 58.62% similar to previous findings (13, 45). Surprisingly, our ICT-IgM exhibited a sensitivity of 75.86% and a specificity of nearly 90%. The PPV and NPV were 81.48% and 85.42%, respectively. Compared to existing IgM assays, the sFlaB-ICT-IgM offers improved diagnostic specificity without compromising sensitivity during the acute phase. This enhancement is attributed to the use of sFlaB, a 50-amino acid fragment derived from the central variable region of FlaB, which improves antigenic specificity by excluding conserved domains that are commonly responsible for cross-reactivity and false positives in conventional serological assays. These results suggest that our ICT-IgM could be highly effective for screening leptospirosis during the acute phase of infection. The sFlaB-ICT not only enhances diagnostic accuracy but also offers a rapid, standardized, and field-deployable alternative that is well-suited for use in resource-limited, endemic settings.

In contrast, when ICT-IgG was tested with sera from the acute phase, the sensitivity and specificity of the test were only 68.97% and 71.4%, respectively. This is because IgG antibody is typically stimulated to produce 1–2 weeks after infection. However, the sensitivity of ICT-IgG did not improve when tested with convalescence-phase sera. This limitation may be attributed to the possibility that sFlaB does not optimally capture IgG antibodies generated during the convalescent phase of the immune response.  

In convalescent serum, ICT-IgM demonstrated a sensitivity of 88.24% and a specificity of 70.59%, while ICT-IgG showed a sensitivity and specificity of 76.47%. The efficiency of ICT in convalescent serum is not much better than in acute serum.

However, this study has some limitations. An appropriate window period for ICT testing is not identified due to the lack of clinical data. The date after the onset of illness when the acute sera were collected should be recorded. In addition, other febrile illness serum samples were not identified. These sera were taken from suspected leptospirosis patients diagnosed according to the clinical manifestations and negative IFA results. Therefore, the ICT test could not indicate the cross-reactivity of other febrile disease infections. To address these issues, further studies should involve kinetic serum samples with well-documented onset of symptoms. Additionally, testing sera from known febrile illness infections will help to identify potential cross-reactivity. This will enable clinicians to interpret results more accurately in conjunction with patient symptoms.

In conclusion, the developed ICT-IgM is effective for screening leptospirosis using a single serum sample of the early phase of infection. Although the sensitivity and specificity of ICT are comparable to those of IFA, ICT offers significant advantages in terms of rapidity and ease of use. It can be performed outside the laboratory and is suitable for field studies or use in suburban laboratories. This rapid detection method is valuable for quickly screening leptospirosis, facilitating early diagnosis and treatment to reduce the severity of the disease.
